# Scapulothoracic Dissociation With Polytrauma Following a High-Velocity Motor Vehicle Collision

**DOI:** 10.7759/cureus.97602

**Published:** 2025-11-23

**Authors:** Mikail Amod, Alana Williams, Tylah Biggs

**Affiliations:** 1 Department of Trauma Surgery, Groote Schuur Hospital, Cape Town, ZAF; 2 Department of Critical Care, Groote Schuur Hospital, Cape Town, ZAF

**Keywords:** forequarter amputation, major limb amputation, motor vehicle accident, polytrauma patient, post operative rehab and soft tissues injuries, scapulothoracic dissociation

## Abstract

Scapulothoracic dissociation (STD) is a rare, limb- and life-threatening injury after high-energy trauma and is frequently obscured by concomitant injuries. This case report details a young adult male with a left open STD following a high-velocity motor vehicle collision. The patient was assessed promptly and managed according to Advanced Trauma Life Support principles with rapid hemodynamic stabilization and early cross-disciplinary coordination, and proceeded to emergency forequarter amputation as part of the damage-control strategy. Postoperatively, he received integrated critical care and a structured, team-based rehabilitation program. This case emphasizes the central importance of early recognition, expeditious resuscitation, a coordinated multidisciplinary approach to optimize trauma outcomes, and early, comprehensive rehabilitation to support recovery after a devastating injury and surgery.

## Introduction

Scapulothoracic dissociation (STD) is defined as a traumatic disruption of the articulation between the scapula, upper extremity, and thorax following traction and blunt-force mechanisms [1,2]. Although uncommon, an STD is often overlooked or mismanaged because it coexists with other severe, life-threatening injuries in complex polytrauma patients [3]. It is often associated with vascular injury, usually involving the subclavian or axillary arteries, as well as brachial plexus injury, fractures of the shoulder girdle, and soft-tissue damage [3]. The Zelle classification stratifies injury severity from type I to IV, with type IV (complete brachial plexus avulsion) representing the most severe and functionally debilitating form [3].

Because of its rarity, no standardized treatment algorithm exists [1]. Initial management should prioritize stabilization according to advanced trauma life support (ATLS) principles with an emphasis on early, aggressive resuscitation [1]. Definitive management depends on the extent of neurovascular and soft-tissue damage. When limb salvage is not feasible because of ischemia, severe brachial plexus injury, or extensive soft-tissue destruction, amputation or disarticulation may be lifesaving [3,4].

Outcomes in reported STD cases are poor, with a mortality of roughly 10% to 11% [3,5]. Given the paucity of cases, each report adds meaningful insight into recognition and management. This case aims to contribute to the literature, emphasizing prompt diagnosis and the need for clear, pragmatic treatment pathways for an STD.

## Case presentation

A young adult, right-hand-dominant male, presented after a high-velocity side-impact motor vehicle collision as an unrestrained driver. He was found unresponsive at the scene and was intubated by emergency medical personnel prior to transfer. On arrival, documented injuries included severe traumatic brain injury, multiple right-sided facial fractures, a left pneumothorax with pulmonary contusions, and an open left STD. A left intercostal drain was in situ, cervical spine immobilization was maintained, and active bleeding from the left shoulder region was compressed.

Initial assessment and resuscitation were conducted in accordance with ATLS principles. The patient arrived mechanically ventilated (field induction with propofol and ketamine). Peripheral intravenous access and a right internal jugular central venous catheter were placed for crystalloid and blood administration, and a left femoral arterial line was placed for invasive monitoring. Pupils were equal and sluggishly reactive. The Glasgow coma scale (GCS) on arrival was recorded as 2T/15 following intubation. A brief episode of pulselessness required approximately two minutes of cardiopulmonary resuscitation with return of spontaneous circulation. In the trauma front room, he received two units of packed red cells and two units of fresh frozen plasma as part of an early balanced transfusion. The extended focused assessment with sonography in trauma (eFAST) was negative, the intercostal drain functioned appropriately, and the abdomen was soft and non-distended.

**Figure 1:**
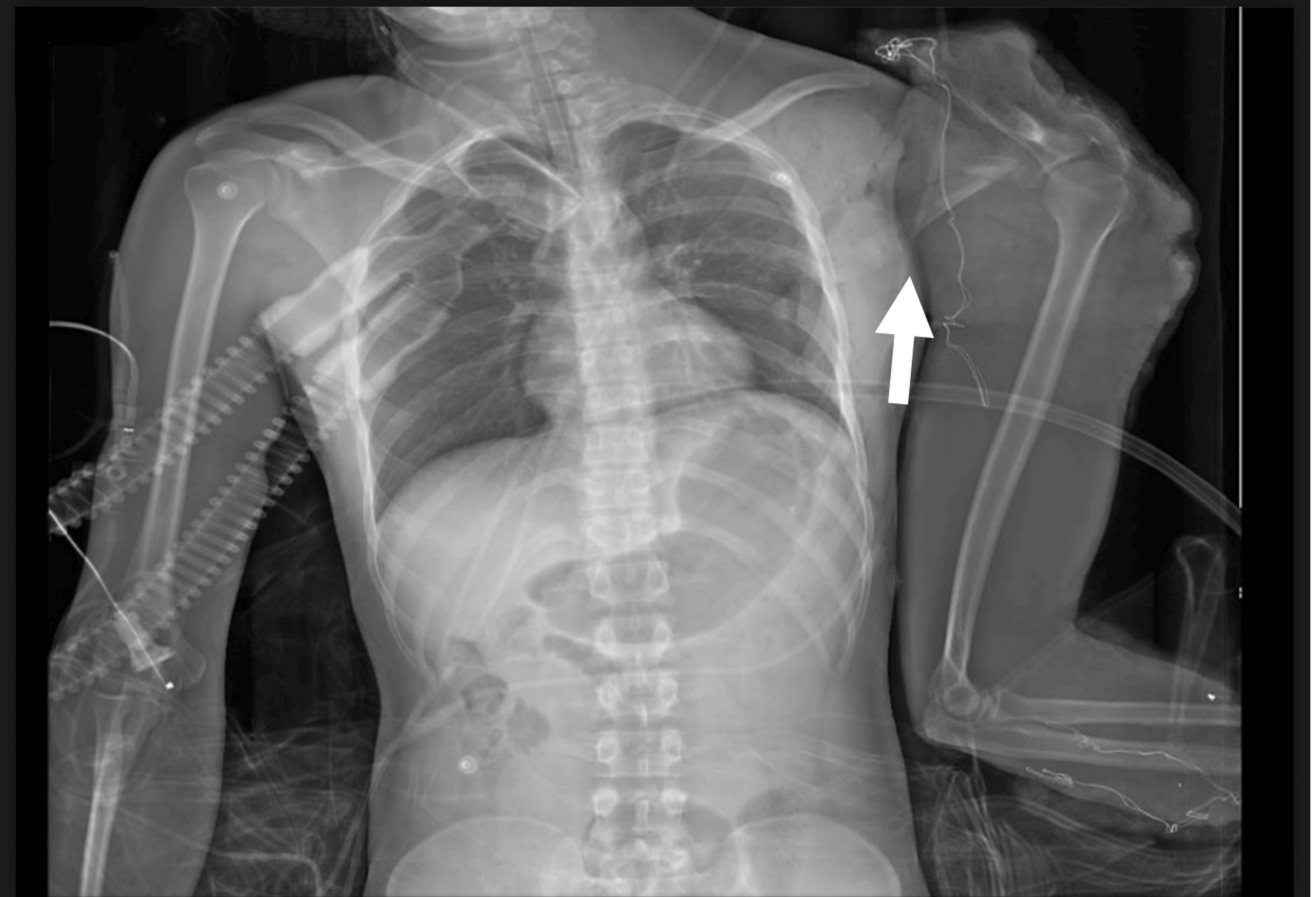
Radiograph (Lodox) on admission showing lateral displacement of the left scapula from the thoracic wall (arrow), consistent with open STD. STD: Scapulothoracic dissociation

**Figure 2:**
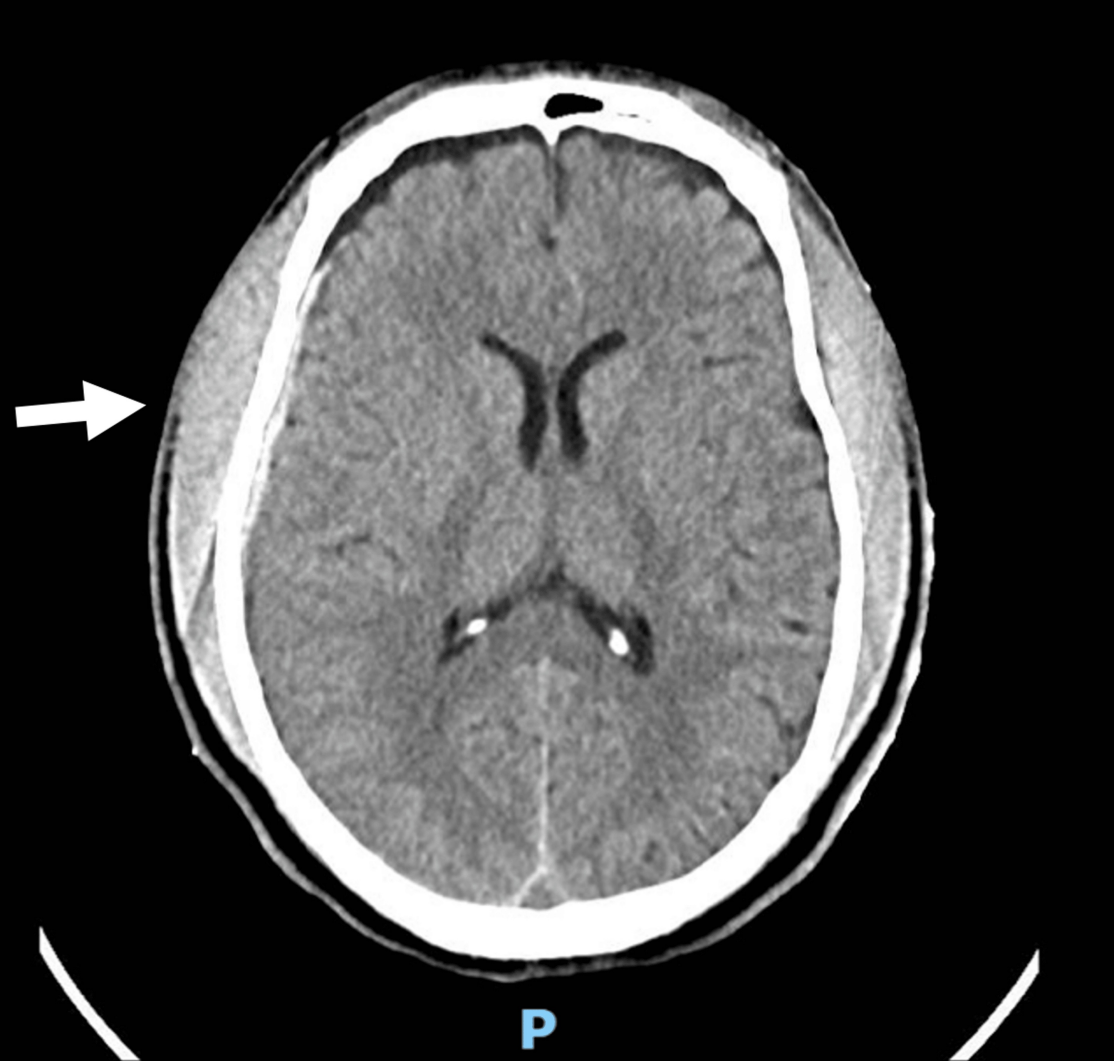
Non-contrast CT brain at presentation demonstrating right frontotemporal subdural haematoma (arrow)

Imaging demonstrated the extent of injury. Low-dose full-body radiography (Lodox; Lodox Systems, Johannesburg, ZAF) showed a left pneumothorax with a deeply positioned intercostal drain, a comminuted left scapular fracture, and findings consistent with an open STD (Figure 1); no pelvic or spinal fractures were seen. A non-contrast CT brain at presentation revealed diffuse injury with a right frontotemporal subdural hematoma (Figure 2). A CT of the thorax and shoulder showed extensive soft-tissue disruption with lateral displacement of the left scapula from the thoracic wall, a comminuted scapular fracture, and separation of the scapula from the chest cage, consistent with an open STD.

Laboratory studies were compatible with acute traumatic blood loss and systemic inflammation. Hemoglobin fell from 10.2 g/dL on admission to 6.7 g/dL and increased to 9.9 g/dL after transfusions. White-cell counts were initially elevated, decreased, and then rose again with reactive thrombocytosis, attributed to post-traumatic inflammatory response and transfusion. Renal function, electrolytes, calcium, magnesium, and phosphate remained within normal limits. Screening for refeeding-related abnormalities during ICU admission was negative (Table 1).

**Table 1:** Blood test results performed at hospital admission compared with preoperative blood results, ICU blood results, and blood results on hospital discharge

Blood test	Reference range	Value at time of hospital admission	Preoperative blood results	ICU blood results	Value at time of hospital discharge
Sodium	136-145 mmol/L	138 mmol/L	136 mmol/L	141 mmol/L	136 mmol/L
Potassium	3.5-5.1 mmol/L	4.2 mmol/L	4.1 mmol/L	4.4 mmol/L	4.6 mmol/L
Urea	2.1-7.1 mmol/L	3.8 mmol/L	4.9 mmol/L	2.9 mmol/L	6.5 mmol/L
Creatinine	64-104 µmol/L	88 µmol/L	122 µmol/L	82 µmol/L	61 µmol/L
White cell count	3.92-10.40 cells x10^9^L	14.26 cells x10^9^L	8.66 cells x10^9^L	13.40 cells x10^9^L	9.56 cells x10^9^L
Hemoglobin	13.0-17.0 g/dL	10.2 g/dL	6.7 g/dL	9.9 g/dL	11.1 g/dL
Platelet count	171-388 Cells x10^9^L	209 Cells x10^9^L	180 Cells x10^9^L	308 Cells x10^9^L	188 Cells x10^9^L
Calcium	2.15-2.50 mmol/L		2.14 mmol/L	2.07 mmol/L	2.20 mmol/L
Magnesium	0.63-1.05mmol/L		0.61 mmol/L	0.68 mmol/L	0.71 mmol/L
Phosphate	0.78-1.42mmol/L		0.81 mmol/L	1.37 mmol/L	1.08 mmol/L

**Figure 3:**
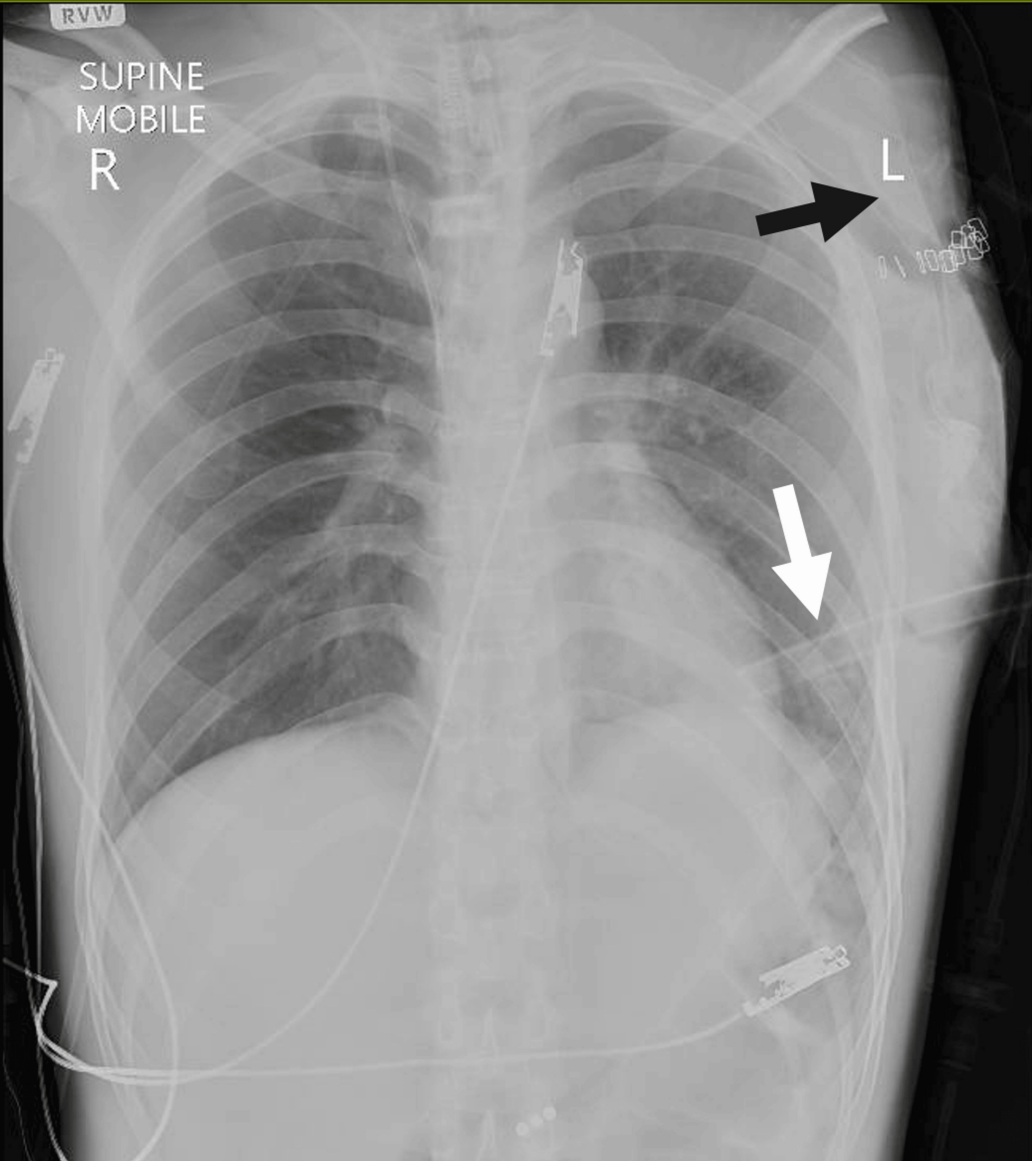
Postoperative chest radiograph White arrow: The intercostal drain position and the absence of immediate thoracic complications following forequarter amputation; Black arrow: Skin staples at the stump site

Given the open, devascularized shoulder girdle injury and the imaging diagnosis of STD with a comminuted scapular fracture, the patient proceeded to emergency left forequarter amputation under general anesthesia on hospital day zero. The operating team comprised an orthopedic surgeon and a registrar assisting. Intravenous co-amoxiclav was administered prophylactically. Intraoperative findings included a comminuted scapular fracture, distal clavicle fracture, and thrombosis of the axillary artery and vein with severe disruption of the deltoid, pectoralis major, serratus anterior, and latissimus dorsi. Proximal control of the axillary vessels was obtained, thrombotic segments were excised, and the axillary artery and vein were ligated. Non-viable muscle was debrided, cords of the brachial plexus were ligated, a 2 cm resection of the distal clavicle was performed, and the wound was irrigated and closed in layers over a drain. Estimated blood loss was approximately 100 mL, and there were no intraoperative complications. A postoperative control chest radiograph confirmed intercostal drain position and no immediate thoracic complication (Figure 3).

Postoperatively, the patient was admitted to the surgical ICU for monitoring, antibiotic prophylaxis, analgesia, thromboprophylaxis, and wound surveillance. He required short-term vasopressor support with adrenaline that was weaned shortly after surgery. A transient metabolic acidosis resolved with intravenous fluids. He self-extubated on postoperative day one and maintained adequate spontaneous ventilation without re-intubation. Enteral feeding via nasogastric tube was tolerated, prophylactic antibiotics for facial fractures were continued, and no clinical sepsis was observed in the ICU. On ICU discharge, the GCS was E3V4M5 (12/15), with eye opening limited by facial pain. An interval non-contrast CT of the brain several days later demonstrated resolution of the right frontal subdural hematoma with no new intracranial hemorrhage.

**Figure 4:**
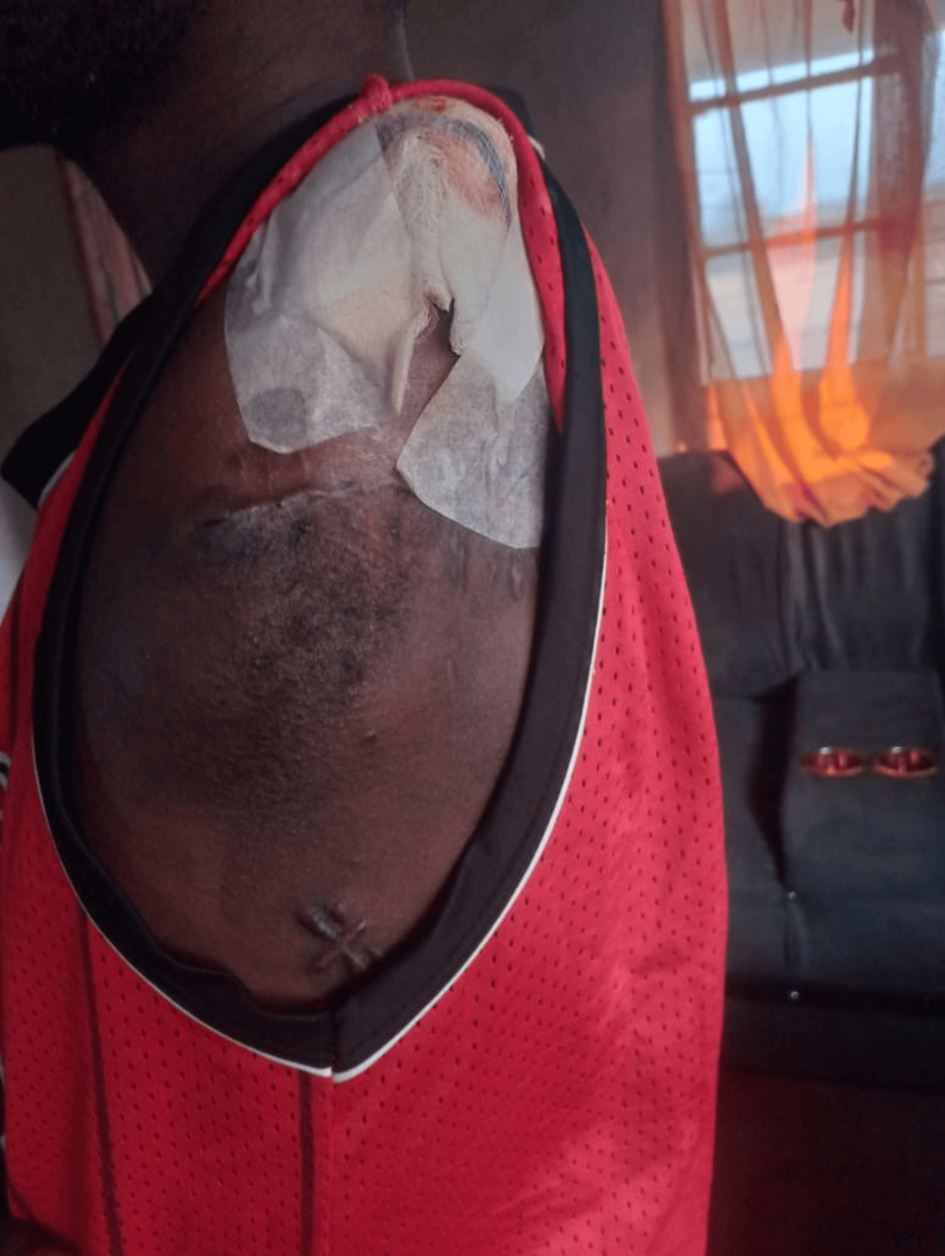
Two-month review photograph demonstrating a well-healed amputation stump without local complication

After two days in the ICU, he remained an inpatient for a further 18 days in the trauma ward, recovering to a GCS of 15/15 and receiving orthopedic follow-up, wound care, physiotherapy, serial blood monitoring, and psychosocial support. The amputation wound healed without complication, and the patient was discharged home in stable condition. Multidisciplinary rehabilitation planning began during admission. He received counseling regarding prosthetic and physiotherapy options, with outpatient follow-up arranged through the orthopedic service. At two months, the stump was well-healed, and he had appropriately engaged with the rehabilitation team (Figure 4).

## Discussion

Scapulothoracic dissociation is a rare, catastrophic shoulder girdle injury defined by disruption of the scapula from the thoracic wall, often with combined osseous, vascular, and neurological damage that is limb-threatening as well as potentially life-threatening [6,7]. It typically follows high-energy deceleration with lateral traction across the shoulder girdle, producing lateral scapular displacement from disruption of scapulothoracic articulation [6]. It is frequently accompanied by brachial plexus disruption and subclavian or axillary vessel injury, and series and reviews report approximately 10% to 11% mortality in severe cohorts, with neurovascular severity largely determining limb viability and long-term function [3,6]. Although STD arises from high-energy mechanisms, presentations are usually with intact overlying skin; an open STD is less common and largely described in isolated reports [3]. The true incidence of STD remains unknown, as published data is limited to isolated case reports and small retrospective case series with no population-level epidemiology. Our case aims to add to the literature by detailing an open STD with concomitant polytrauma (traumatic brain injury, facial fractures, and chest injury) typical of high-velocity events.

Modern classification, often cited as the Zelle modification of the Damschen system (Table 2), stratifies STD by musculoskeletal disruption plus the presence and severity of vascular and neurologic injury, with type IV denoting complete brachial plexus avulsion and the worst prognosis [3,6]. In this patient, the combination of an open STD with a comminuted scapular fracture and a non-reconstructable upper limb culminating in forequarter amputation is clinically consistent with type IV severity, which is associated with limited prospects for limb salvage [3,8]. In polytrauma, a screening chest radiograph may suggest STD via lateral scapular displacement, but CT angiography (CTA) is recommended as the first-line vascular study because it expedites definitive management compared with conventional angiography [9]. A pragmatic workflow is ATLS resuscitation followed by chest radiography if feasible (scapular index as a clue), then urgent CTA to delineate subclavian and axillary injury and guide operative planning, after which decisions regarding revascularization or amputation are made [10]. The vascular injury profile on CTA, together with clinical signs of a nonviable limb (devitalized tissue, uncontrollable contamination, or hemorrhage), informs the early decision between attempted revascularization and primary amputation in STD [6].

**Table 2:** Table showing summary of the Zelle classification of STD Summary of the injury severity categories described in the Zelle classification system [3]. STD: Scapulothoracic dissociation

Type	Description
Type I	Isolated musculoskeletal injury
Type II A	Musculoskeletal injury with vascular disruption
Type II B	Musculoskeletal injury with incomplete brachial plexus injury
Type III	Musculoskeletal injury with both vascular disruption and incomplete brachial plexus injury
Type IV	Musculoskeletal injury with complete brachial plexus injury; represents the most severe form

All trauma patients’ initial management should be as per ATLS principles, prioritising airway protection, breathing and ventilation (including tube thoracostomy for pneumothorax), and circulation with hemorrhage control and damage control resuscitation, before proceeding to limb-specific decisions [11]. Immediate limb-focused priorities are hemorrhage control and contamination control, with early broad-spectrum antibiotics and tetanus prophylaxis, followed by prompt, generous surgical debridement and hemostasis before any definitive decisions on salvage versus amputation [6,12]. Definitive decision making hinges on the CTA defined vascular injury pattern and clinical limb viability: when there is non-reconstructable subclavian or axillary disruption, gross devitalization or contamination, prolonged warm ischemia, or suspected complete brachial plexus injury, primary forequarter amputation is appropriate; otherwise a staged damage control approach (debridement, shunt or repair, fasciotomies, later definitive reconstruction) may be attempted [6]. When primary forequarter amputation is selected, the operation entails en bloc removal of the upper limb with the shoulder girdle (scapula with or without the clavicle), with proximal control and ligation of the subclavian and axillary vessels, radical debridement, and myocutaneous flap closure tailored to contamination and soft tissue loss [13].

Functional outcome in STD is driven chiefly by the severity of brachial plexus injury, with complete plexus disruption predicting a nonfunctional limb even if revascularised, and compounded by the extent of associated major vascular injury [6,8]. Following forequarter amputation, rehabilitation is focused on activities of daily living (stump care, pain control, graded independence) because functional prosthetic use at very proximal levels is limited; upper limb prosthesis rejection and abandonment are substantial, and many patients ultimately forgo a functional device in favour of non-prosthetic adaptation or cosmetic options [13,14].

After upper limb amputation, phantom limb pain and residual limb pain are common, so early multimodal analgesia and expectation setting and education should be integrated into the rehabilitation plan [15,16]. Where available, targeted muscle reinnervation, ideally performed at the time of amputation, can reduce neuroma and phantom limb pain and may facilitate myoelectric prosthesis control in selected patients [17]. Major upper limb loss carries a substantial psychological and social burden; screening for depression and post-traumatic stress disorder, early referral to psychology, and coordinated vocational rehabilitation should be integrated into the care pathway alongside physical rehabilitation [18]. In this instance, our patient was reviewed by the multidisciplinary team, including occupational therapy, psychology, and physiotherapy, and was provided with a comprehensive, staged rehabilitation programme.

## Conclusions

Scapulothoracic dissociation remains an uncommon but devastating consequence of high-velocity trauma, and our case underscores the extreme presentation of an open injury with a nonviable limb consistent with type IV severity. Early ATLS-based resuscitation with decisive CTA-guided triage enabled prompt primary forequarter amputation, limiting operative time and facilitating stabilization despite concomitant traumatic brain, facial, and chest injuries. Equally critical, early and sustained rehabilitation paired with structured psychological counseling supports pain management, functional retraining, and adaptation to daily life, which are central to long-term recovery after major upper limb loss. This experience reinforces a pragmatic pathway: recognize STD early, use CTA to define vascular injury, proceed decisively when salvage is futile, and engage rehabilitation and psychosocial support at the earliest safe opportunity.
